# Mitochondrial disease and epilepsy in children

**DOI:** 10.3389/fneur.2024.1499876

**Published:** 2025-01-09

**Authors:** Xuan Zhang, Bo Zhang, Zhiming Tao, Jianmin Liang

**Affiliations:** ^1^Department of Pediatric Neurology, Children's Medical Center, First Hospital of Jilin University, Changchun, China; ^2^Jilin Provincial Key Laboratory of Pediatric Neurology, Changchun, China; ^3^Neuromedical Center, First Hospital of Jilin University, Changchun, China

**Keywords:** epilepsy, mitochondrial complex, coenzyme Q, cytochrome C, genes

## Abstract

Mitochondria is the cell’s powerhouse. Mitochondrial disease refers to a group of clinically heterogeneous disorders caused by dysfunction in the mitochondrial respiratory chain, often due to mutations in mitochondrial DNA (mtDNA) or nuclear DNA (nDNA) that encodes mitochondrial proteins. This dysfunction can lead to a variety of clinical phenotypes, particularly affecting organs with high energy demands, such as the brain and muscles. Epilepsy is a prevalent neurological disorder in children and is also a frequent manifestation of mitochondrial disease. The exact mechanisms underlying epilepsy in mitochondrial disease remain unclear and are thought to involve multiple contributing factors. This review explores common mitochondrial diseases associated with epilepsy, focusing on their prevalence, seizure types, EEG features, therapeutic strategies, and outcomes. It also summarizes the relationship between the molecular genetics of mitochondrial respiratory chain components and the development of epilepsy.

## Highlights

- The incidence of epilepsy in children with mitochondrial disease is higher than in adults.
- The pathogenesis of mitochondrial-associated epilepsy in children is complex, involving various genetic mutations.
- Understanding the molecular genetics of mitochondrial respiratory chain components can aid in diagnosing and treating epilepsy for diagnosis and treatment.
- While no cure currently exists for mitochondrial diseases, gene therapy is a promising potential treatment option.

## 1 Mitochondrial physiology and mitochondrial diseases

Mitochondria, essential organelles in eukaryotic cells, produce energy by generating adenosine triphosphate (ATP) through oxidative phosphorylation (OXPHOS). This energy production occurs within the mitochondrial respiratory chain (MRC) situated in the inner mitochondrial membrane. The MRC consists of five enzyme complexes (complexes I to V) encoded by both mitochondrial DNA (mtDNA) and nuclear DNA (nDNA), along with two electron transport proteins, coenzyme Q10, and cytochrome C, which are both encoded by DNA. Therefore, mitochondrial diseases can result from both maternally inherited mtDNA mutations and classical genetic mutations in nDNA (1).

Dysfunction of the MRC in mitochondrial diseases is often caused by mutations in mtDNA or nDNA. mtDNA mutations include point mutations, deletions, duplications, and depletion of copy number. nDNA mutations impact several processes, such as the formation of MRC subunits, assembly factors, intergenomic signaling, mitochondrial protein synthesis, lipid metabolism, mitochondrial dynamics (including fusion and fission), and coenzyme Q10 homeostasis (2). ATP is essential for all cells, but MRC dysfunction disproportionately affects those with high energy demands, such as neurons, skeletal muscles, and retinal ganglion cells. Beyond energy production, mitochondria play key roles in intracellular calcium regulation, reactive oxygen species (ROS) production, apoptosis, and neurotransmitter synthesis.

## 2 Mechanism of epilepsy caused by mitochondrial dysfunction

Epilepsy, a prevalent neurological disorder, partly arises due to the central nervous system’s substantial energy demands. When mitochondrial function is compromised, energy deficits can precipitate seizures. Zsurka et al. suggest that the pathogenesis of mitochondrial-associated epilepsy involves processes such as neuronal energy depletion, oxidative stress, impaired calcium signaling, neurotransmitter imbalances, *β*-oxidation deficiency, and glial cell dysfunction (3). Additional factors, such as changes in cerebral blood flow, structural abnormalities in the brain, and immune-mediated damage, also contribute to the development of mitochondria-related epilepsy.

Mutations in specific mtDNA or nDNA genes encoding the mitochondrial respiratory chain are closely related to epilepsy. These gene mutations prevent the oxidative phosphorylation process in the mitochondria from proceeding normally, resulting in a decrease in intracellular ATP levels. This disruption increases neuronal excitability by damaging the activity of sodium and potassium ATP enzymes and decreasing membrane potential (4). After the occurrence of mitochondrial-associated epilepsy, the energy demand of neurons that have been metabolically damaged increases. This energy exhaustion will further lead to the production of reactive oxygen species (ROS), apoptosis, and abnormal calcium homeostasis, which will promote epilepsy, thus forming a vicious circle (5). ROS is mainly produced in mitochondria. High concentrations of ROS can oxidize mitochondrial proteins, lipids, and nucleic acids, cause cell dysfunction, damage the ability of cells to maintain energy levels, cause energy exhaustion, damage and death of neurons, and change the excitability of neurons, thus reducing the threshold of seizures (6, 7). The imbalance of calcium homeostasis caused by mitochondrial dysfunction can increase the excitability of neurons and induce seizures. Levetiracetam can play a partial antiepileptic effect by regulating the level of intracellular calcium (8). The relative imbalance of excitatory and inhibitory neurotransmitters may cause seizures. Disorders of receptor and ion channel gene mutation/protein function lead to abnormal stimulus transmission and epileptic discharges. Disorders of neurotransmitters such as glutamate, *γ*-aminobutyric acid (GABA), acetylcholine (Ach), dopamine, 5-hydroxytryptamine, norepinephrine, histamine, melatonin, and nitric oxide are involved in the pathogenesis of epilepsy (9). Epilepsy is often accompanied by neuronal overexcitation. Repeated seizures can cause oxidative stress, inflammation, and excitotoxic damage and eventually lead to neuronal death, including apoptosis, autophagy, necrotizing apoptosis, scorch death, and iron death (10, 11). The changes in ion channels, transporters, and metabolism of astrocytes are also closely related to the occurrence of epilepsy (9). Astrocytes are rich in mitochondrial glutamate carrier SLC25A22, which controls glutamate uptake. Its functional loss will lead to extracellular glutamate imbalance and activation of extrasynaptic glutamate receptors, increase neuronal excitability, and cause epilepsy (12, 13). Congenital metabolic defects, such as fatty acid *β* oxidation disorder, make it impossible for the liver to use fatty acids as a source of energy, resulting in a decrease in blood glucose levels (14). Hypoglycemia results in seizures through excitatory neurotoxicity mediated by the N-methyl-D-aspartate receptor (NMDAR), increasing the production of mitochondrial free radicals, initiating apoptosis, and changing brain energy production (15).

**Figure 1:**
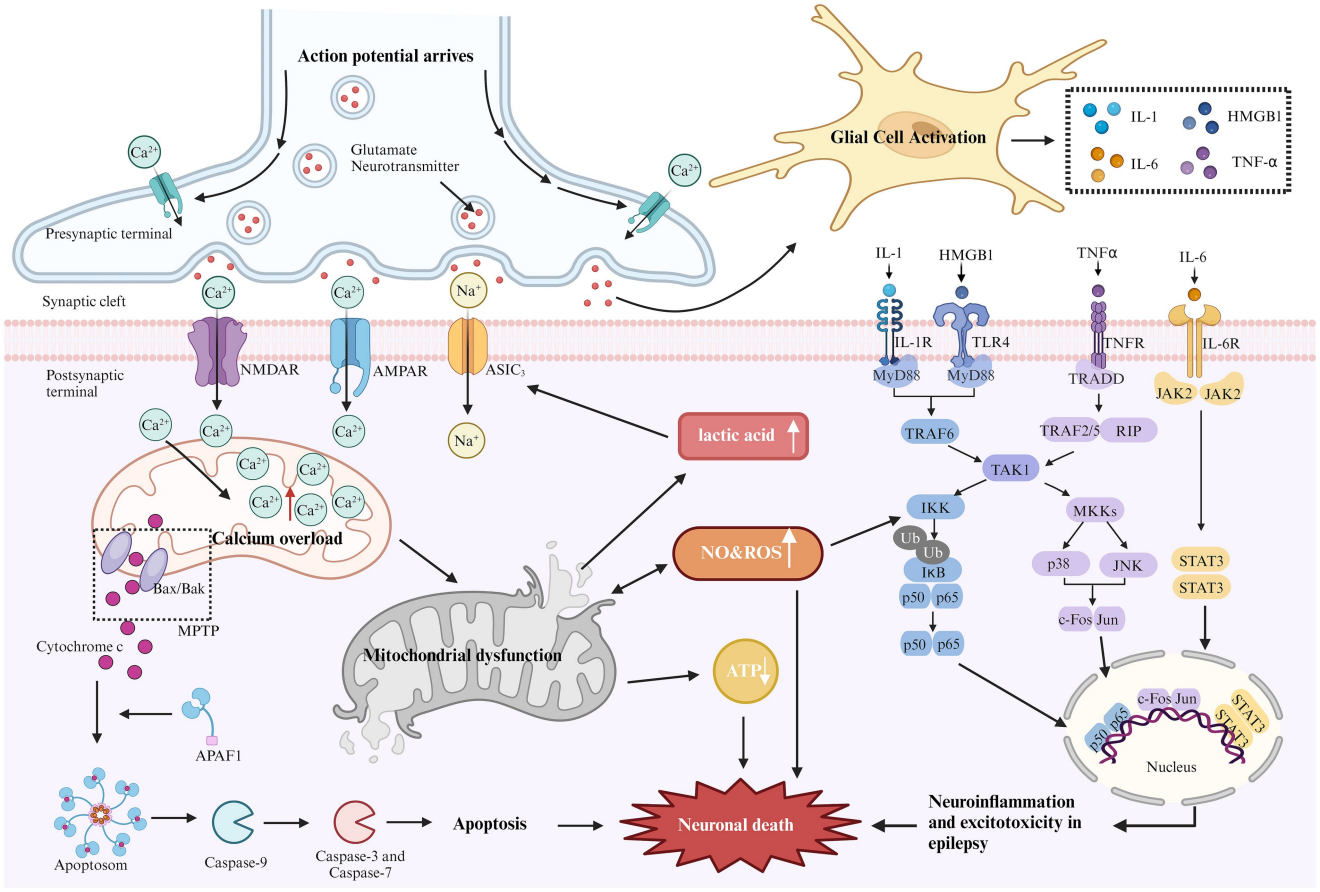
Mechanism of epilepsy-related cell death. During epilepsy, excitability increases due to the loss of inhibitory neurons. The elevated glutamate levels or mutations in metabolic receptors (e.g., AMPA, NMDA) can prolong the activation time of glutamatergic receptors, increase excitatory postsynaptic potential, lead to excitotoxicity, and trigger downstream apoptotic cascades. Excessive activation of NMDA and AMPA receptors can cause a sharp influx of sodium and calcium ions. Excessive calcium influx leads to calcium overload in the mitochondrial matrix, resulting in mitochondrial dysfunction. This dysfunction opens the mitochondrial permeability transition pore (MPTP), leading to the release of cytochrome C into the cytoplasm. Cytochrome C binds with apoptosis-related factor 1 (APAF1), promoting caspase-9 binding to form apoptosomes, thereby activating other caspases and initiating the cascade of apoptosis, ultimately inducing neuronal cell death. Continuous brain discharges increase energy demand and decrease oxygen levels, leading to increased anaerobic glycolysis. Lactic acid, as a by-product of anaerobic glycolysis, appears to activate acid-sensitive ion channels (ASIC3), causing further calcium and sodium ion influx, which aggravates excitotoxicity. In addition, mitochondrial dysfunction reduces ATP production and increases reactive oxygen species (ROS) generation. Long-term seizures often activate the innate immune response, leading to glial cell activation and promoting the release of cytokines (e.g., IL-1, HMGB1, TNF-α, and IL-6). These cytokines activate several inflammatory pathways, such as NF-kB, p38, JNK, and JAK–STAT, regulating the expression of pro-inflammatory mediators and transcription factors, resulting in neuronal apoptosis.

Energy deficiency can stimulate the proliferation of mitochondria of smooth muscle and small vascular endothelial cells and cause angiopathy (such as MELAS syndrome), resulting in damage to microvascular hemoperfusion and insufficient energy supply to the brain (16). ATP deficiency can cause astrocyte dysfunction and excitotoxicity, leading to nerve cell death. In addition, hemodynamics and metabolic stress enhance the mobilization of nitric oxide (NO) and decrease the level of circulating NO. Both mechanisms are involved in epilepsy (17). Mitochondrial disease-related epilepsy may also be related to immune dysfunction (18). Recently, it has been reported that MELAS syndrome is complicated by autoimmune injury, but the mechanism between it and autoimmune disease is not clear and needs further study (19). The RARS2 gene is the pathogenic gene of cerebellopontine dysplasia type 6 (PCH6). MRI is characterized by cerebellopontine dysplasia or progressive cerebellopontine and cerebellar cortex atrophy (20). MT-TL1 gene mutations in mtDNA can involve the cortex, mostly located in the occipital lobe and parietal lobe, and may be accompanied by cerebellar and cerebellar atrophy (21). Brain structural abnormalities caused by RARS2 and MT-TL1 gene mutations are common seizures. It can be seen that the cause of brain structural venereal disease is also one of the pathogenesis of mitochondrial-related epilepsy. The pathways of cell death associated with epilepsy are shown in Figure 1. The mechanisms of epilepsy caused by various mitochondrial diseases are different, and understanding its underlying pathological mechanism is very important to develop reasonable strategies for the treatment of mitochondrial-related epilepsy.

Although the mechanisms of epilepsy vary across different mitochondrial diseases, Figure 1 outlines common pathways of cell death associated with epilepsy. Understanding these underlying pathological mechanisms is crucial for developing effective strategies to treat mitochondrial-related epilepsy.

## 3 Mitochondrial diseases and epilepsy in children

Mitochondrial diseases in children can present at any age and often affect multiple systems. In these conditions, seizures tend to originate in the occipital lobe or posterior brain regions, with seizure types ranging from epileptic spasms (as seen in infantile epileptic spasm syndrome), focal seizures (with or without secondary generalization), generalized seizures, myoclonic seizures, and refractory status epilepticus (22). In pediatric patients, myoclonic seizures are the most frequently observed seizure type associated with mitochondrial diseases (23). The incidence of epilepsy in children with mitochondrial disease ranges from 25 to 100%, in contrast to approximately 14% in adults. Children with mitochondrial-related epilepsy exhibit a higher prevalence of focal seizures and nDNA mutations compared to adults. Their clinical presentation aligns more closely with electro-clinical syndromes and mitochondrial disease-related syndromes (24).

Mitochondrial diseases can be genetically categorized based on mutations in mtDNA or nDNA, which encode mitochondrial proteins. mtDNA mutations are implicated in various disorders, including mitochondrial encephalomyopathy with lactic acidosis and stroke-like episodes (MELAS), maternally inherited Leigh syndrome (MILS), myoclonic epilepsy with ragged red fibers (MERRF), Leber’s hereditary optic neuropathy (LHON), Kearns-Sayre syndrome, Pearson syndrome, and chronic progressive external ophthalmoplegia (CPEO). In contrast, nuclear gene mutations encode mitochondrial proteins, resulting in a more complex pathogenic mechanism. Although the incidence of nDNA mutations in mitochondrial disease is lower, they are associated with conditions such as Leigh syndrome, Alpers-Huttenlocher syndrome (AHS), GRACILE syndrome, and Bjornstad syndrome. A wide variety of these mitochondrial disorders are associated with epilepsy, as outlined below.

## 4 MELAS and epilepsy

The pathogenesis of MELAS is associated with gene mutations, vascular disease, nitric oxide (NO) dysregulation, and energy deficiency. The most commonly implicated genetic mechanism is the mutation of the *MT-TL1* gene, with the most frequent mutation being m.3243A > G (25). In MELAS, seizures occur with a frequency ranging from 71 to 96% (26), and typically present as generalized tonic–clonic seizures, focal seizures, or generalized status epilepticus (27).

Electroencephalograms (EEGs) in patients with MELAS typically show diffuse background slowing, often accompanied by slow waves mixed with sharp waves, especially in the bilateral anterior regions (28). The most common EEG findings are non-specific diffuse or focal slow-wave activity in the bilateral occipital and anterior head regions. Epileptic discharges are typically focal but may become widespread and are frequently associated with seizures (27). Regardless of the genotype or presence of status epilepticus, about 30% of patients exhibit transient, non-specific, periodic unilateral epileptic discharges during the acute phase, though these are rarely observed in the chronic phase (29). Additionally, some MELAS patients experience loss of fixation sensitivity (FOS), an EEG phenomenon in which epileptic discharges are triggered by loss of central vision or gaze, characterized by idiopathic or generalized epileptic discharges following eye closure for more than 3 s (30).

**Figure 2:**
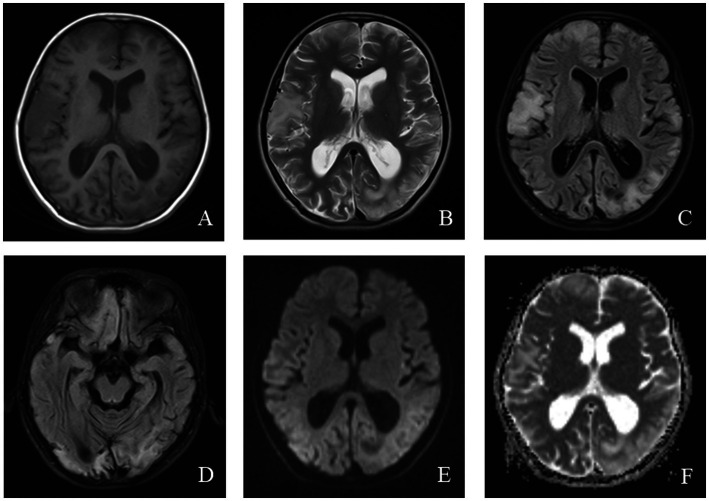
An 11-year-old female was diagnosed with MELAS syndrome due to intermittent convulsions for 3.5 years, which worsened over the last 7 days. Head MRI revealed: **(A)** Low signal intensity in the bilateral frontal, parietal lobes on axial T1WI. **(B–D)** On axial T2WI and T2/FLAIR, the brain tissue of the bilateral frontal parietal-occipital lobes was swollen, showing strip-like and gyrus-like high signal intensity. **(E)** DWI showed high signal intensity in the gyrus of the lesion area. **(F)** High signal intensity of the corresponding lesions was observed on the ADC.

Magnetic resonance imaging (MRI) in MELAS patients typically reveals cortical lesions, primarily in the occipital and parietal lobes, presenting as low signal intensity on T1-weighted imaging (WI) and high signal intensity on T2WI. These lesions do not follow the typical vascular distribution of cerebral arteries and may be accompanied by cerebellar or cerebral atrophy (21). Newer imaging modalities, such as oxygen uptake fraction measurements, transcranial Doppler ultrasound, and magnetoencephalography, can provide additional insight into the affected brain tissue and aid in prognosis prediction (31). MRI findings from a pediatric MELAS patient are shown in Figure 2.

## 5 LS and epilepsy

Leigh syndrome (LS), also known as subacute necrotizing encephalomyelopathy, is associated with approximately 100 different mtDNA or nDNA gene defects (32). LS can result from mitochondrial tRNA mutations in Cox I, II, III, IV, V, and PDH. Based on the age of onset, LS is classified into early-onset (before 2 years of age) and late-onset (after 2 years). A meta-analysis of 385 LS cases revealed that nDNA mutations were more common than mtDNA mutations (38% vs. 32%), and earlier onset of symptoms was more frequently associated with nDNA mutations. Additionally, 80% of patients had defects in respiratory chain enzyme complexes, with 35% displaying isolated complex I defects (33).

The typical clinical presentation of LS includes motor retardation, seizures, reduced exercise tolerance, and lethargy (34). Seizures are the second most common symptom, with 13.3% of cases initially manifesting as epilepsy (35). A report of 110 LS cases from Korea found that focal seizures with impaired consciousness were the most prevalent seizure type, followed by generalized myoclonic, tonic, atonic, and tonic–clonic seizures. While seizure types did not significantly differ between early- and late-onset LS, focal and generalized tonic–clonic seizures were most common in late-onset cases (34, 36).

EEG findings in LS are variable. Most patients exhibit a decreased and disorganized background rhythm, along with focal or multifocal sharp waves during the interictal period (37). In one case of late-onset LS, diffuse spike–wave complexes at 3 ~ 4 Hz were observed (38). Another case of LS caused by an *MT-ND1* mutation demonstrated decreased background activity, multifocal epileptic discharges, and significant irregularity (39).

**Figure 3:**
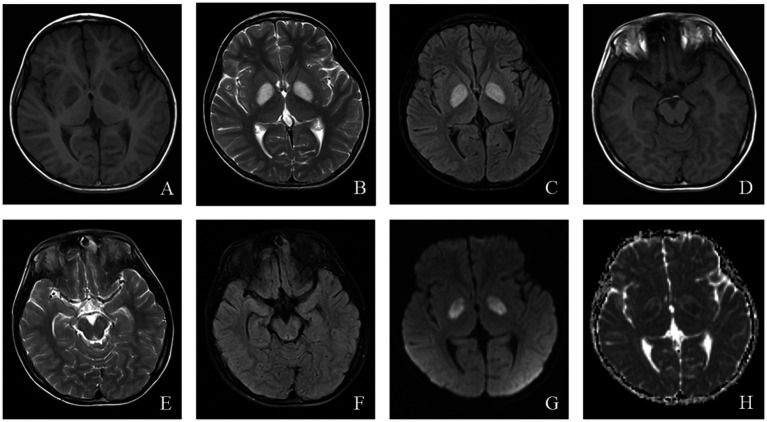
An 8-year-old male who experienced intermittent fever and limb weakness for over 6 years and drowsiness for 1 day was diagnosed with Leigh syndrome. Head MRI showed the following: **(A–F)** On axial T1WI, patchy low signal intensity was seen in the bilateral globus pallidus and midbrain, while patchy high signal intensity was observed in the bilateral globus pallidus and midbrain on axial T2WI and T2/FLAIR. **(G)** High signal intensity in the focal area was observed on DWI. **(H)** ADC showed high and low signal intensity in the corresponding lesions.

MRI findings in LS often help elucidate the underlying etiology, with classic abnormalities involving the basal ganglia, particularly the bilateral putamen, periaqueductal gray, and brainstem (40). In patients with *SURF-1* mutations, symmetrical involvement of the bilateral brainstem and subthalamic nuclei is common, and some patients exhibit basal ganglia abnormalities. In contrast, patients without *SURF* mutations show symmetrical bilateral basal ganglia involvement without significant white matter or brainstem abnormalities (41). The MRI findings of a pediatric patient with LS are shown in Figure 3.

## 6 AHS and epilepsy

AHS is an autosomal recessive genetic disorder caused by biallelic mutations in the *POLG* gene, which encodes a DNA polymerase responsible for the replication and repair of mtDNA. These mutations result in mitochondrial dysfunction, including mtDNA mutations, deletions, or depletion. The most common mutation associated with AHS is A467T (42, 43).

Approximately 50% of children with AHS present with focal, multifocal, or myoclonic seizures at the onset of epilepsy. As the disease progresses, most patients develop recurrent seizures, including status epilepticus and persistent focal epilepsy (epilepsia partialis continua, EPC) (44, 45). EPC is a hallmark feature of AHS, and in some cases, multi-site EPC is observed early, aiding in early diagnosis.

In AHS, EEGs typically show reduced background activity along with high-amplitude slow waves in the occipital region. This is often accompanied by occasional spike–wave or intermittent to continuous spike–wave or multi-spike–wave activity, known as rhythmic high-amplitude delta with superimposed (poly)spikes (RHADS) (44). The presence of RHADS in patients with status epilepticus may indicate AHS (46). If RHADS is not detected on the initial EEG, extended monitoring may increase the likelihood of identification. RHADS generally appears in the early stages of AHS and is not affected by anti-seizure medications (ASMs). It is frequently associated with high-energy gamma (*γ*) oscillations, which may reflect active epileptic lesions (47).

**Figure 4:**
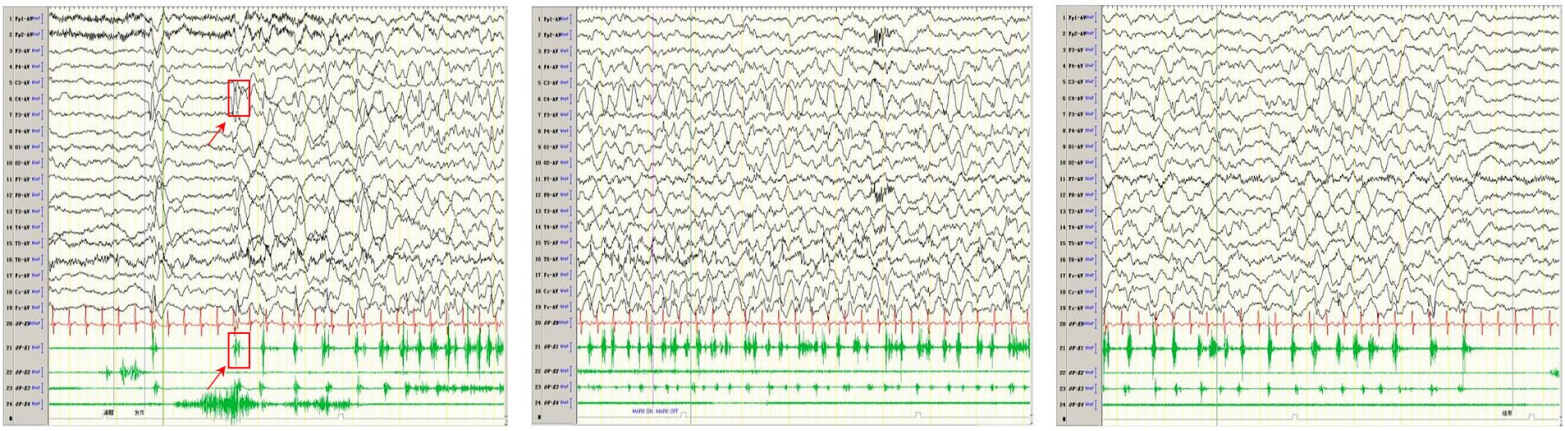
In one patient with Alpers syndrome, EEG showed a 2-3 Hz slow-wave complex (multiple) spike sign in the right central region, that is, the characteristic rhythmic high-amplitude *δ* (RHADS) of superposition (multiple) spike waves, and simultaneous left-side limb jitter (EPC). One time of jitter is shown in the arrow and box in the figure. The top arrow indicates the EEG performance, and the bottom arrow indicates the same myoelectric performance. Each subsequent jitter is the same as this one.

**Figure 5:**
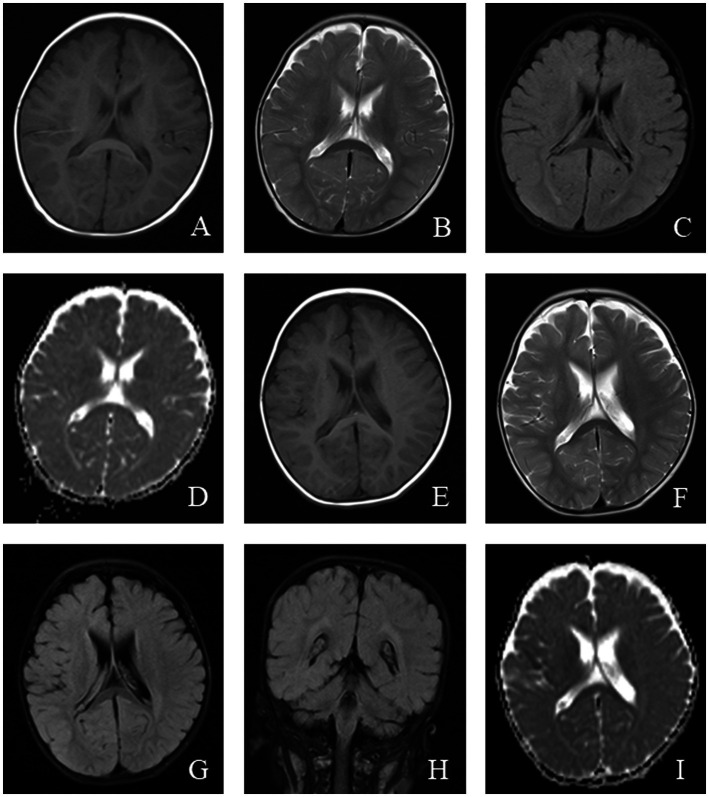
An 11-month-old male was diagnosed with Alpers syndrome due to persistent left limb convulsions for 4 h. Initial head MRI showed: **(A–C)** No abnormal signals in T1WI, T2WI, and dark-fluid images, small ventricles, and no displacement of midline structures in the brain tissue. **(D)** Bilateral symmetrical low signal intensity was observed on the ADC map of the caudate nucleus. Reexamination after 3 months: **(E–H)** Patchy abnormal signals were observed around the bilateral ventricles. T1WI showed iso-signal, while T2WI and fluid-attenuated inversion recovery (FLAIR) images showed slightly high signal intensity. **(I)** Bilateral symmetrical low signal intensity was still visible on the ADC images of the caudate nucleus.

Computed tomography (CT) in AHS typically reveals low-density areas in the gray and white matter of the posterior temporal and occipital lobes, which may progress to diffuse cerebral atrophy in later stages (48). MRI shows high signal intensity on T2WI or FLAIR sequences, particularly in the deep nuclei of the occipital lobes, thalamus, basal ganglia, and cerebellum (49). The EEG and MRI findings from a pediatric patient with AHS are illustrated in Figures 4, 5.

## 7 MERRF and epilepsy

MERRF is a rare mitochondrial disorder caused by pathogenic mutations in mtDNA. Approximately 80% of cases are attributed to the m.8344A > G point mutation in the *MT-TK* gene, leading to reduced complex I and IV activity, decreased respiratory rate and compromised mitochondrial membrane potential (50). This mutation can also result in an overlap syndrome involving MELAS, MERRF, and LS.

MERRF affects multiple systems and typically presents with myoclonus as the initial symptom, followed by generalized seizures, ataxia, fatigue, motor intolerance, and dementia. Seizure frequency in MERRF patients varies widely, ranging from 33 to100% (51). Generalized myoclonic seizures are the most common, followed by focal atonic seizures, focal clonic seizures, generalized tonic–clonic seizures, myoclonic absence seizures, typical absence seizures, and status epilepticus (52, 53). There is ongoing debate regarding whether the motor symptoms in MERRF represent myoclonic seizures or cerebellar or spinal cord symptoms, with some researchers proposing the term “myoclonic ataxia” rather than “myoclonic epilepsy” (51).

EEGs in MERRF patients lack specificity but are generally characterized by background slowing, generalized epileptic discharges, and focal epileptic discharges. The EEG patterns may vary depending on the specific mutation. For example, patients with the m.8344A > G mutation in MT-TK may exhibit widespread sharp waves, sharp-slow wave complexes, or slow waves, while those with the m.3291 T > C mutation show widespread or focal multi-spike slow waves. Similarly, the m.4279A > G mutation can produce extensive spike–wave or multi-spike slow-wave complexes (51).

**Figure 6:**
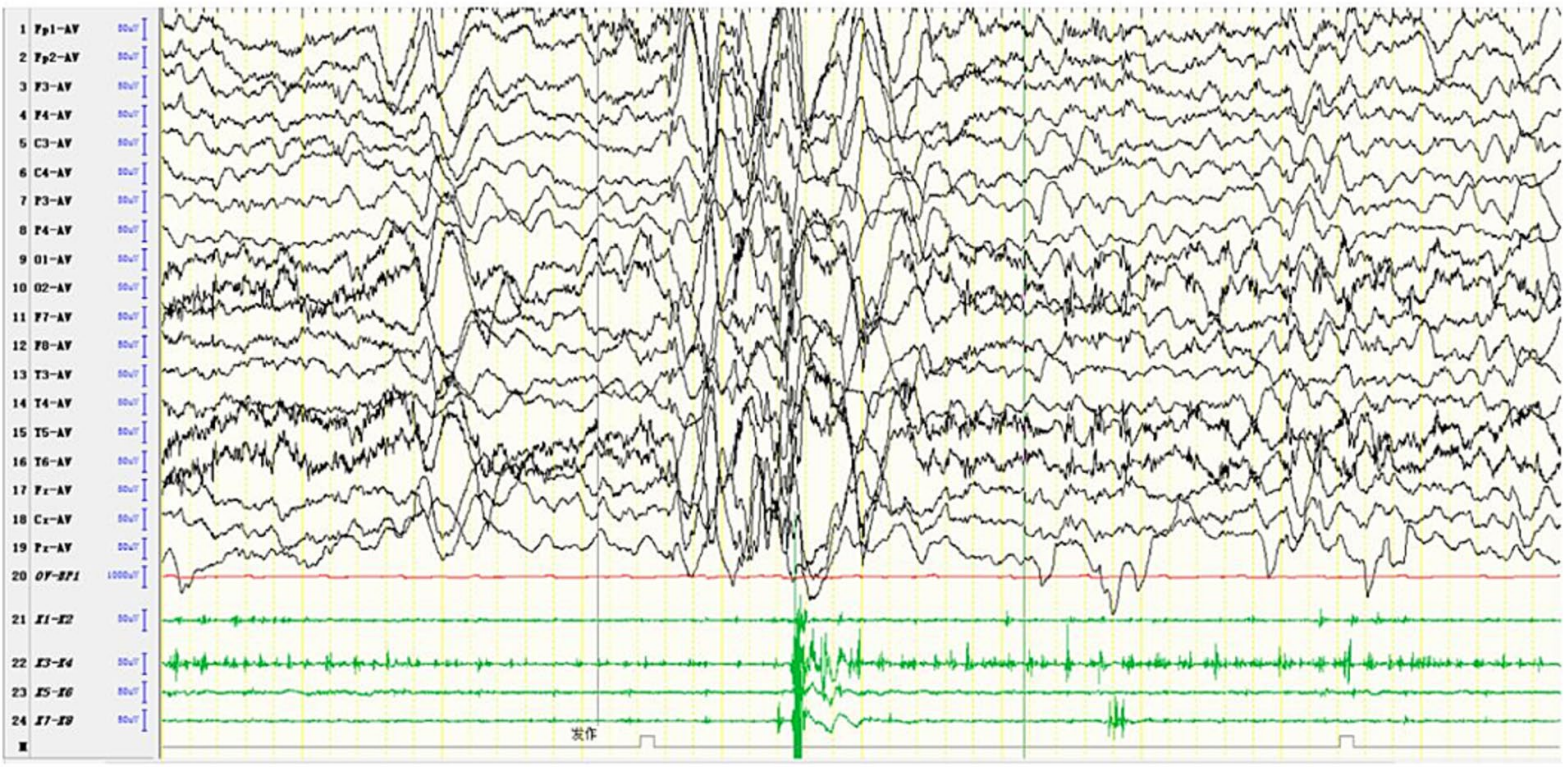
In one patient diagnosed with MERRF, an episode was detected during the awake period, showing limb tremors. The EEG showed widespread 3–4 Hz moderate to high-amplitude spike and slow waves for 1–2 s, with a transient EMG burst lasting 200 ms occurring simultaneously.

**Figure 7:**
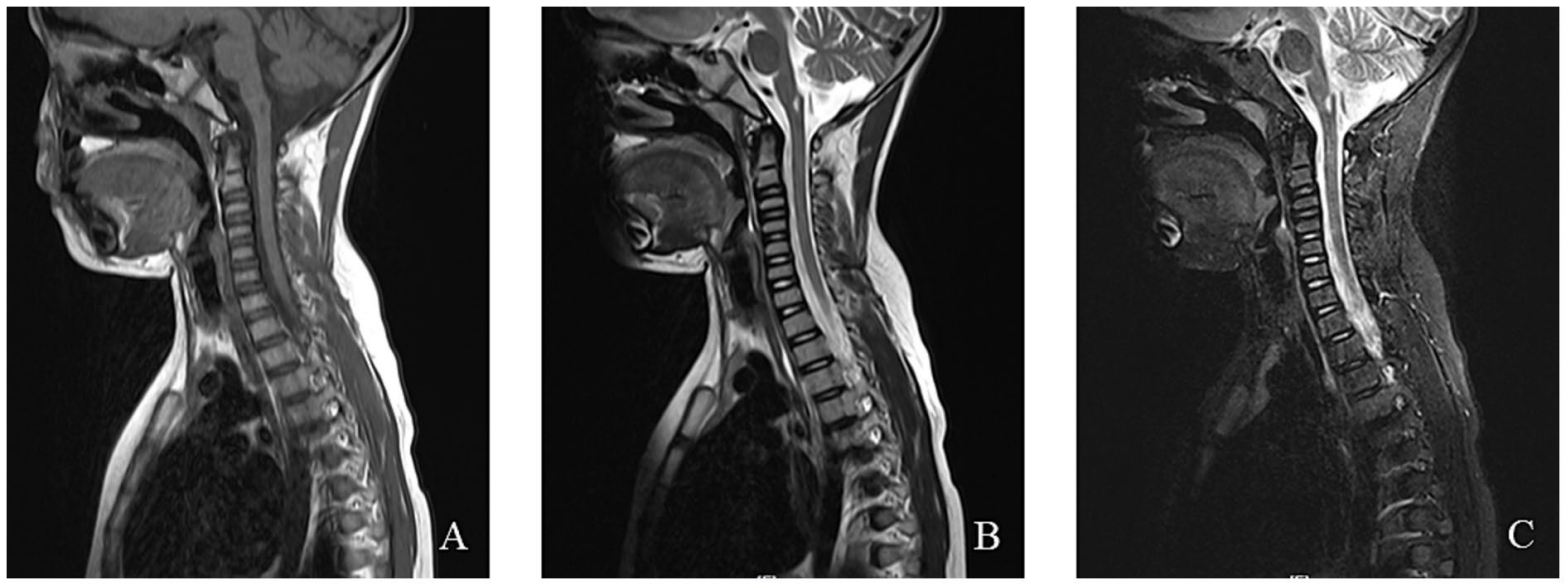
An 8-year-old female was diagnosed with MERRF syndrome due to limb tremors for more than 10 months. **(A–C)** Cervical MRI showed a patchy abnormal signal in the medulla oblongata. T1WI showed a low signal, T2WI showed a high signal, and the fat-suppression image showed a slightly high signal.

MRI findings in MERRF often reveal cerebellar and cerebral atrophy, focal white matter abnormalities, and bilateral symmetrical lesions in the brainstem, subthalamic nuclei, and basal ganglia (54). MRS frequently shows a decreased cerebellar N-acetyl aspartate/creatine ratio, with no significant increase in lactate levels (55). The EEG and MRI findings from a pediatric MERRF patient are shown in Figures 6, 7.

## 8 Epilepsy characteristics caused by different mitochondrial complex dysfunctions

**Figure 8:**
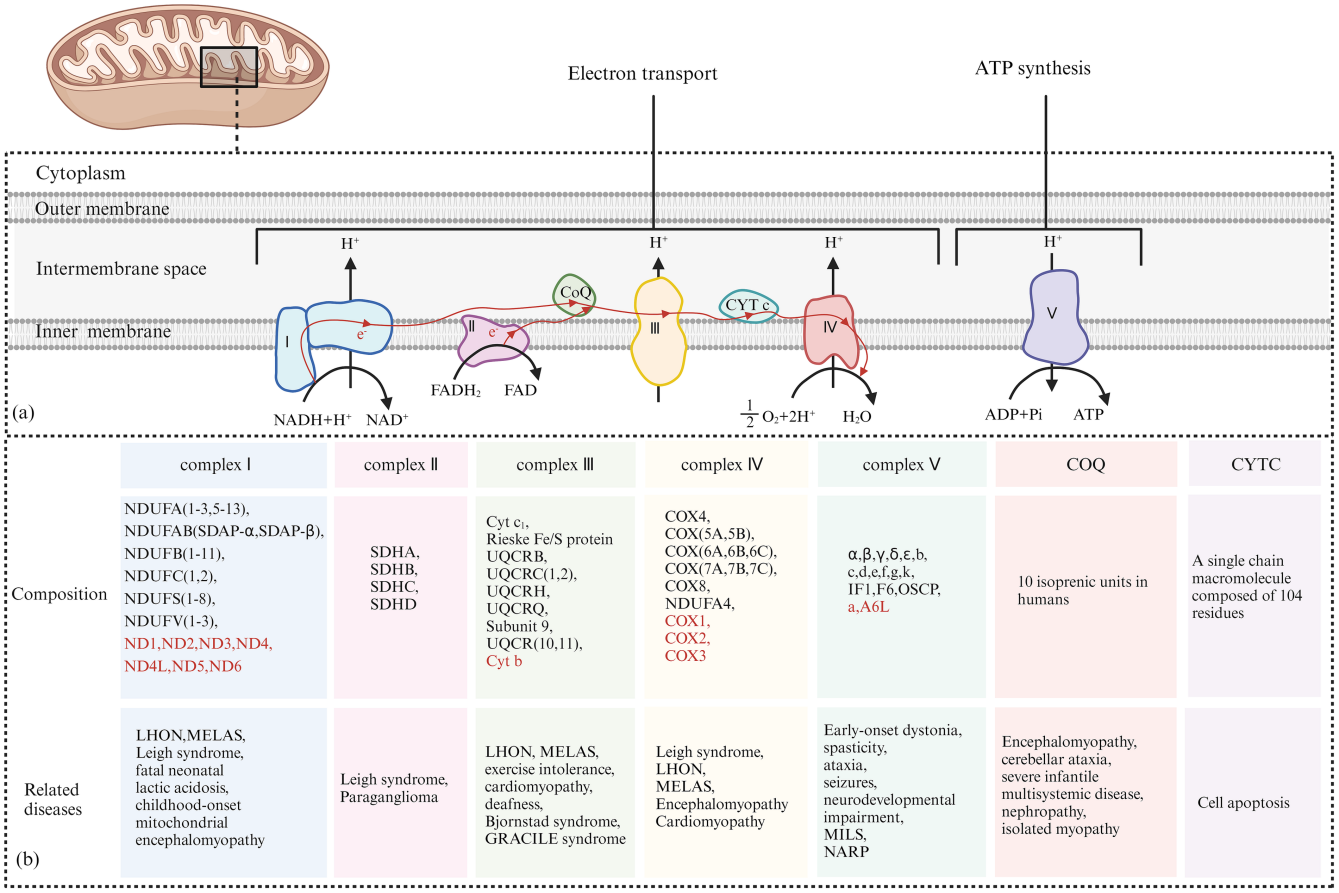
**(A)** Electron Transfer Process of the Mitochondrial Respiratory Chain: Complex I receives electrons from NADH + H^+^ and transfers them to coenzyme Q. The released energy pumps H^+^ from the matrix side of the mitochondria to the intermembrane side. Complex II receives electrons from FADH₂ and transfers them to coenzyme Q, but the energy released during this electron transfer is insufficient to pump H^+^ out. Coenzyme Q collects electrons from Complex I and Complex II, and the resulting QH₂ shuttles to Complex III. Complex III transfers electrons from QH₂ to CytC through a “Q cycle.” The energy released during the electron transfer in Complex III can pump H^+^ from the matrix side of the mitochondria to the intermembrane side. Complex IV receives electrons from CytC and transfers them to O₂ to produce H₂O, and the energy released during this electron transfer can also pump H^+^ to the intermembrane side. The H^+^ pumped into the intermembrane space forms an electrochemical gradient of H^+^, which is transformed into a proton-motive force, driving H^+^ back into the matrix along the concentration gradient and releasing stored potential energy. This stored energy is fully utilized by Complex V to catalyze the formation of ATP from ADP and Pi. **(B)** The Composition of the Five Complexes of the Mitochondrial Respiratory Chain and Two Types of Electron Transport Proteins (Coenzyme Q10 and Cytochrome C): In addition, typical cases of diseases caused by their respective genetic mutations are presented. The sources of each subunit are classified according to whether they are encoded by mitochondrial or nuclear genes. Subunits encoded by nuclear DNA (nDNA) are marked in red, while subunits encoded by mitochondrial DNA (mtDNA) are marked accordingly.

The classification of mitochondrial diseases is based on several factors, including the affected tissues and organs, the dysfunction of respiratory chain enzyme complexes, and the types of gene mutations involved. The MRC consists of five enzyme complexes, along with two key electron transport proteins: coenzyme Q10 and cytochrome C. These components are essential for the proper functioning of the electron transport chain (ETC). Figure 8 illustrates the five MRC complexes and the composition of the electron transport proteins while listing common diseases caused by mutations in these complexes. Additionally, Table 1 presents the mitochondrial and nuclear genes that encode the proteins of the mitochondrial electron transport chain.

**Table 1:** Summary of the genes, seizure types, and EEG characteristics of mitochondrial disease-related epilepsy.

Pathogenesis		Gene location	Genetic modality	Coding gene	Seizure types	Electroencephalogram (EEG) characteristics	Onset time	Treatments	Outcomes
OXPHOS subunit defects	Complex I	mt DNA	Mat	*MT-ND1 MT-ND3 MT-ND4 MT-ND5 MT-ND6*	*MT-ND3*: focal motor, persistent focal, generalized clonic, persistent focal, visual sensitivity, and generalized tonic–clonic seizures (57, 58, 60). *MT-ND6*: myoclonic seizures, generalized tonic–clonic seizures (65) *MT-ND1:* focal seizures (142) *MT-ND5:* focal seizures (143) *MT-ND4:* unclear	EEG shows sharp waves, spikes in certain areas, extensive slow-wave activity, and periodic slow and sharp waves, with sharp waves present in some brain regions.	Birth to school age	Antiepileptic drugs, cocktail therapy, etc.	Most patients die within 10 years, but a small number of patients can survive to adulthood.

n DNA	AR, XL	*NDUFA1 NDUFA2 NDUFA9 NDUFA10 NDUFA11 NDUFA12 NDUFA13 NDUFB3 NDUFB9 NDUFB11 NDUFS1 NDUFS2 NDUFS3 NDUFS4 NDUFS6 NDUFS7 NDUFS8NDUFV1 NDUFV2*	*NDUFA1:* tonic clonic seizures, generalized seizures (144) *NDUFA2:* generalized tonic–clonic seizures (145) *NDUFA9:* focal seizures (146) *NDUFV1:* myoclonic seizures (147) *NDUFS4:* generalized seizures (148) *NDUFA8 NDUFA10 NDUFA11 NDUFA12 NDUFA13 NDUFB3 NDUFB9 NDUFB11 NDUFS1 NDUFS2 NDUFS3 NDUFS6 NDUFS7 NDUFS8 NDUFV2:* seizures may occur, but the type of seizure is unknown

Complex II	n DNA	AR	*SDHA SDHB SDHC SDHD*	*SDHA:* myoclonic seizure (73), Focal and generalized seizures (74), epileptic spasms (75) *SDHB:* febrile seizures (72) *SDHD:* generalized tonic–clonic seizures, multifocal focal seizures, myoclonic seizures (77). *SDHC:* unclear	The EEG findings show nonspecific slow-wave activity and focal epileptic discharges (74).	It occurs from infancy to adulthood, but is more common in infants and young children.	High-dose riboflavin therapy	Most of the neurological symptoms persist.

Complex III	mt DNA	Mat	*MT-CYB*	spastic seizures, status epilepticus, focal non-convulsive status epilepticus, or generalized tonic–clonic seizures (83–85).	EEG findings showed decreased background activity and occasional generalized epileptic discharges	School-age	Antiepileptic drugs, cocktail therapy, etc.	Unclear

n DNA	AR	*UQCRC2*	generalized seizures (80)	Unclear	Infancy, Toddlerhood	Symptoms may be mild

Complex IV	mt DNA	Mat	*MT-CO1 MT-CO2 MT-CO3*	*MT-CO1:* myoclonic seizure, generalized tonic–clonic seizures, focal seizures, status epilepticus (101, 102, 149) *MT-CO2 MT-CO3:* unclear	Electroencephalograph (EEG) recordings revealed focal or diffuse epileptiform activities and marked background slowing. The ictal EEG discharges associated with partial motor seizures had frontocentral location, but generalized sharp wave paroxysms were also observed (149).	School Age, Adolescence	Antiepileptic drugs, cocktail therapy, and others.	Residual neurological symptoms

n DNA	AR	*COX4l1 COX6B1 COX8A NDUFA4*	*COX4l1:* epileptic spasms (104) *COX6B1:* generalized tonic–clonic seizures (150) *COX8A:* focal and generalized tonic–clonic seizures, myoclonic seizures atonic seizures (151) *NDUFA4:* generalized tonic–clonic seizure (152)	EEG varies by seizure type	Birth to school age

Complex V	mt DNA	Mat	*MT-ATP6 MT-ATP8*	*MT-ATP6:* focal seizures, generalized tonic–clonic seizures (GTCS), myoclonic seizures, atonic seizures, progressive myoclonus epilepsy (PME) (111, 113, 115) *MT-ATP8:* generalized clonic seizures (117)	From slow background activity to variable combinations of paroxysmal/epileptiform activities (111).	From fetal period to 75 years old.	Antiepileptic drugs	Most affected children present with an overall homogeneous Leigh or Leigh-like syndrome phenotype

n DNA	AR	*ATP5F1E ATP5PO*	*ATP5F1E:* generalized tonic–clonic seizures (108) *ATP5PO:* focal seizures (108)
OXPHOS assembly factor defects	Complex I	n DNA	AR	*NDUFAF1 NDUFAF2 NDUFAF3 NDUFAF4 NDUFAF5 NDUFAF6 FOXRED1 ACAD9 NUBPL C17orf89*	*NDUFAF3:* myoclonic seizures (153) *NDUFAF6:* focal seizures (154) *NDUFAF1 NDUFAF2 NDUFAF4 NDUFAF5 FOXRED1 ACAD9 NUBPL C17orf89:* seizures may occur, but the type of seizure is unknown	EEG varies by seizure type	Birth to school age	Antiepileptic drugs, cocktail therapy, etc.	Most patients die within 10 years, but a small number of patients can survive to adulthood.

Complex II	n DNA	AR	*SDHAF1 SDHAF2*	Unclear	Unclear	Birth to infancy	High-dose riboflavin therapy	Most of the neurological symptoms persist.

Complex III	n DNA	AR	*BCS1L HCCS LYRM7/MZM1L TTC19 UQCC2*	*UQCC2:* generalized seizures and status epilepticus (90, 91).	EEG findings showing low-voltage activity (90, 91).	Infancy	Mechanical ventilation and other life support treatments	Death in infancy

Complex IV	n DNA	AR	*SCO1 SCO2 COX10COX15 SURF1 PET100 FASTKD2 LRPPRC*	*SCO1:* generalized clonic seizures, generalized tonic–clonic, generalized motor seizures (155) *SCO2:* focal seizure (98) *SURF1:* generalized tonic clonic seizure, myoclonic seizure (96) *PET100:* tonic seizures (156) *FASTKD2:* focal status epilepticus, generalized tonic clonic seizure, focal to bilateral tonic clonic seizure (157, 158) *LRPPRC, COX10, COX15:* unclear	EEG varies by seizure type	Birth to adulthood	Antiepileptic drugs, cocktail therapy, and others.	Residual neurological symptoms

Complex V	n DNA	AR	*ATPAF2/ATP12 TMEM70*	*TMEM70:* generalized seizures, status epilepticus (119, 159) *ATPAF2/ATP12:* unclear	Unclear	Birth to early childhood	Antiepileptic drugs	Unclear
Coenzyme Q		n DNA	*AR*	*COQ1-PDSS2 COQ2 COQ4 COQ5 COQ6 COQ8-ADCK3 COQ8-ADCK4 COQ9*	*COQ2:* focal seizures with altered consciousness, persistent partial seizures, myoclonic seizures, and status epilepticus (122, 134) *COQ4:* status epilepticus, myoclonic seizures, and focal seizures (126) *COQ8:* focal seizures, generalized tonic–clonic seizures, myoclonic seizures, absence seizures, persistent partial epilepsy, and status epilepticus (129–132).	EEG varies by seizure type	Birth to adulthood	CoQ10 or its derivatives	Some patients die early, while others progress slowly
Cytochrome c		n DNA	Unclear	*CYCS*	Unclear	Unclear	Unclear	Unclear	Unclear

AR, autosomal recessive; Mat, maternal inheritance; XL, X-linked; OXPHOS, oxidative phosphorylation.

### 8.1 Mitochondrial complex I and epilepsy

Mitochondrial complex I, also known as NADH dehydrogenase or NADH-Q reductase, consists of 45 structural subunits. Deficiency in mitochondrial complex I is associated with mutations in various structural subunits and assembly factors, resulting in a wide range of clinical manifestations, including LHON, infantile LS, MELAS, and epilepsy (56).

Among the core subunits of complex I, mutations in *MT-ND3* and *MT-ND5* are more common. Point mutations in MT-ND3, such as m.10191 T > C, m.10158 T > C, and m.10197G > A, have been widely studied. More than 30 patients with the *MT-ND3* m.10191 T > C mutation have been reported, with approximately 90% developing epilepsy, typically between the ages of 8 and 10. Seizure types include focal motor, persistent focal, and generalized tonic–clonic seizures (57, 58).

EEG findings in patients with the *MT-ND3* m.10191 T > C mutation typically display interictal focal epileptic discharges, sharp waves, spikes in certain areas, and extensive slow-wave activity, with sharp waves present in some brain regions (57, 59). Imaging often reveals LS-like changes, such as involvement of the basal ganglia, thalamus, and brainstem, in about 50% of patients, with some exhibiting white matter-dominant polyencephalic lesions (57).

The MT-ND3 m.10158 T > C mutation is occasionally associated with isolated and recurrent stroke-like seizures. These seizures typically occur in middle age or later, manifesting as generalized clonic seizures, persistent focal seizures, and visual sensitivity seizures (60). EEG has periodic slow and sharp waves (61). MRI findings usually indicate lesions in the posterior cortex of the supratentorial area (62).

The common *MT-ND5* m.13513G > A mutation is linked to multiple syndromes, including LS, MELAS, MERRF, LHON, and MELAS-LS overlap syndrome (63, 64). Some patients with this mutation also experience seizures. Dermaut et al. reported that a patient with the *MT-ND6* m.14487 T > C mutation presented with myoclonic seizures, generalized tonic–clonic seizures, decreased EEG background activity, spike waves, and multi-spike waves (65). Patients with *NDUFV1* mutations may exhibit myoclonic seizures and absence seizures (66, 67). Seizures are also linked to mutations in nDNA genes, including *NDUFS2*, *NDUFS4*, *NDUFS8*, *NDUFA1*, and *NDUFA8* (68–70).

Currently, there is no effective treatment for mitochondrial complex I deficiency. Clinical management primarily focuses on enhancing mitochondrial energy metabolism, increasing antioxidant capacity, providing nutritional support, and using ASMs to control seizures. Approximately 60% of patients with the *MT-ND3* m.10191 T > C mutation survive into puberty or later, which is a better prognosis than other complex I-related gene defects. A report from Japan indicated that homologous expression of codon-optimized *MT-ND3* in patients with *MT-ND3* mutations can increase ATP production and improve mitochondrial function, showing promise as a potential new treatment strategy (71).

### 8.2 Mitochondrial complex II and epilepsy

Mitochondrial complex II, also known as succinate dehydrogenase (SDH) or succinate-ubiquinone oxidoreductase (SQR), Comprises four structural subunits: SDHA, SDHB, SDHC, and SDHD, all encoded by nDNA. Additionally, four assembly factors, SDHAF1, SDHAF2, SDHAF3, and SDHAF4, are involved in its assembly and function (72). Clinical manifestations of complex II deficiency vary and include LS, leukoencephalopathy, optic nerve atrophy, and epilepsy. Among 61 patients with complex II deficiency reported by Fullerton et al., five had seizures (72).

Mutations in *SDHA* are the most common cause of complex II deficiency and are mainly associated with LS. Neurological symptoms include epileptic encephalomyopathy, external ophthalmoplegia, and myoclonic seizures (73). In one case of an *SDHA* mutation, the patient experienced focal and generalized seizures, with EEG findings showing nonspecific slow-wave activity and focal epileptic discharges (74). Another case presented with Lennox–Gastaut syndrome and highly irregular EEG patterns (75). Both cases demonstrated imaging features resembling LS.

Biallelic variations in *SDHB* frequently result in complex II deficiency. Approximately 10 patients with LS have been identified with pathogenic *SDHB* variations, most exhibiting neurodegenerative symptoms, seizures, and dystonia, with one patient showing heat sensitivity (72). The clinical manifestations of complex II deficiency related to SDHB mutations are similar to LS but typically do not involve the basal ganglia, which can help differentiate the two conditions (76).

A review by SiyingLin et al. identified six patients with *SDHD* mutations, four of whom experienced generalized tonic–clonic seizures, multifocal focal seizures, and myoclonic seizures (77). Of the four known SDH assembly factors, only mutations in *SDHAF1* have been linked to complex II deficiency. Andreas et al. reported five patients with homozygous *SDHAF1* mutations, all of whom had motor degeneration and spastic paraplegia, with only one patient experiencing seizures (78).

Since complex II deficiency is rarer than other mitochondrial diseases, limited reports exist on its treatment and prognosis. Riboflavin has been shown to improve clinical manifestations in patients with complex II deficiency, potentially preventing disease progression and even reversing symptoms. In particular, patients with riboflavin transporter deficiency may benefit from high-dose riboflavin therapy (up to 70 mg/kg/day) (79).

### 8.3 Mitochondrial complex III and epilepsy

Mitochondrial complex III, also known as ubiquinone-cytochrome c reductase or cytochrome bc1 complex, consists of 11 structural subunits. Cytochrome b is encoded by mitochondrial DNA (mtDNA), while the remaining 10 subunits are encoded by nuclear DNA (nDNA). Additionally, BCS1L, TTC19, and UQCC2, encoded by nDNA, are involved in the assembly of complex III (80). The Leucine zipper EF-hand domain transmembrane protein 1 (LETM1) is located in the inner mitochondrial membrane and plays a critical role in regulating the synthesis of cytochrome b. Downregulation of LETM1 reduces MT-CYB expression and increases susceptibility to seizures (81).

Mutations in cytochrome b can manifest in skeletal muscle involvement, exercise intolerance, MELAS, LS, and seizures (82). Seizures in these cases may present as spastic seizures, status epilepticus, focal non-convulsive status epilepticus, or generalized tonic–clonic seizures (83, 84). Scott et al. described a patient with a cytochrome b mutation who exhibited both Parkinson’s syndrome and MELAS, along with status epilepticus and tonic–clonic seizures. EEG findings showed decreased background activity and occasional generalized epileptic discharges (85).

*BCS1L* mutations are the most common cause of complex III deficiency and are linked to GRACILE syndrome, Björstand syndrome, liver disease, encephalopathy, dyskinesia, and epilepsy. Neurological symptoms, such as dyskinesia and seizures, generally manifest after the age of 1 month, with an epilepsy incidence of 33%. Patients with the BCS1L c.232A > G mutation have typically developed GRACILE syndrome, although epilepsy is often absent (86). Erika et al. described a case of rapid progression in a patient with a *BCS1L* mutation, characterized by growth retardation, epileptic spasms, and frequent seizures in later stages. MRI findings showed involvement of the thalamus and supratentorial white matter (87). Helen et al. described a patient with a novel BCS1L mutation who had slower disease progression, presenting with myopathy, focal motor seizures, and optic nerve atrophy (88). Hikmat et al. reported that 6 out of 33 patients with *BCS1L* mutations had seizures (86).

Mutations in *TTC19*, *UQCC2*, and *UQCRC2* are also associated with seizures. Patients with missense *TTC19* mutations (c.971 T > C and c.554 T > C) may present with tonic seizures and refractory epilepsy (89). Patients with *UQCC2* mutations may experience generalized seizures and status epilepticus, with EEG findings showing low-voltage activity (90, 91). There have also been reports suggesting that mutations in the UQCRC2 gene can lead to generalized seizures (80). MRI findings in patients with complex III defects vary widely. Some resemble those seen in LS, with focal and bilateral symmetrical lesions in the basal ganglia, thalamus, and brain stem, while others show brain atrophy or underdeveloped myelin.

There is no specific therapy for respiratory chain complex III deficiency. Treatment focuses on managing symptoms and slowing disease progression. In one case, clinical symptoms improved following treatment with vitamin K3 and vitamin C (92). Patients with *TTC19* mutations often present with early psychomotor retardation, followed by progressive neurological decline and poor prognosis (89). The survival rate for patients with homozygous or compound heterozygous *BCS1L* c.232A > G mutations is significantly lower compared to patients with other BCS1L mutations, underscoring the importance of genotype–phenotype correlation, prognosis evaluation, and genetic counseling (86). Early disease onset is generally associated with faster progression and higher mortality.

### 8.4 Mitochondrial complex IV and epilepsy

Mitochondrial complex IV, also known as cytochrome c oxidase (COX), is the terminal enzyme in the electron transport chain. It comprises 14 subunits, with 3 encoded by mitochondrial DNA (mtDNA) and the remaining 11 by nuclear DNA (nDNA). Several nuclear-encoded assembly factors, including SURF1, SCO1, SCO2, COX10, COX15, and LRPPRC, are involved in the assembly of complex IV (93). Cytochrome c oxidase deficiency affects the brain, heart, and skeletal muscle, leading to disorders such as LS and cardiomyopathy (94).

*SURF1* deficiency is a monogenic mitochondrial disorder and the most common cause of cytochrome c oxidase-deficient LS. Approximately 80% of *SURF1* mutations are truncating, caused by abnormal splicing, frameshift deletions, or nonsense mutations. Clinical features include weight loss, dystonia, stunted growth, and epilepsy, with an epilepsy incidence of 33.3%, most commonly presenting as generalized tonic–clonic and myoclonic seizures (95). MRI findings often show LS-like features, with symmetrical necrotic lesions in the basal ganglia and brainstem (96). In comparison, patients with *SCO2* mutations tend to have earlier-onset symptoms that progress more rapidly, including hypertrophic cardiomyopathy, dystonia, and seizures. EEG findings commonly show focal epileptic discharges in the central and temporal regions (97, 98). The COX15 p.R217W mutation is associated with lactic acidosis, ataxia, and hypotonia, with epilepsy occurring in 71.4% of cases. Status epilepticus has also been reported, though specific seizure types have not been well-documented. EEG findings typically include multifocal slow-wave discharge (99, 100).

In addition to mutations in assembly factor genes, mutations in the three COX subunits encoded by mtDNA are also linked to epilepsy. *MT-CO1* mutations can lead to a wide range of seizure types, including myoclonic seizures associated with the m.6480G > A and m.6930G > A missense mutations (101, 102). The m.7402delC mutation can cause non-convulsive status epilepticus (102). The missense mutation m.7023G > A is associated with seizures such as mid-wind, generalized tonic–clonic, and focal seizures. Patients with the m.6489G > A mutation display persistent partial and myoclonic seizures, generalized tonic–clonic seizures, recurrent status epilepticus, and diffuse epileptic activity on EEG, often accompanied by significantly reduced background activity. MT-CO3 subunit mutations have been reported in patients with recurrent MELAS-like seizures (103).

In mammals, COX4 has two isoforms: COX4I1 and COX4I2. The *COX4I1* c.454C > A missense mutation has been linked to infantile spasms, high EEG irregularity, and multifocal spikes (104).

Treatment options for mitochondrial complex IV deficiency are limited and vary depending on the specific mutation. Arginine may help alleviate stroke-like episodes in patients with COX-deficient MELAS, while coenzyme Q10 has shown promise in slowing disease progression in COX4I1 deficiency (16, 104). EPI-743, a coenzyme Q10 derivative, has been found to prevent disease progression and improve quality of life and motor function in some *SURF1*-related LS cases (105). Additional treatments, such as the Pan-PPAR agonist bezafibrate and the AMPK agonist AICAR, have shown the potential to partially restore COX function in human and animal models (106). Intrathecal delivery of an adeno-associated viral vector serotype 9 (AAV9)-human SURF1 (hSURF1) has effectively corrected biochemical abnormalities in mouse models of SURF1 deficiency (107). Emerging therapies such as mitochondrial biogenesis and gene replacement also hold promise for future treatment strategies.

### 8.5 Mitochondrial complex V and epilepsy

Mitochondrial complex V, also known as ATP synthase, is essential for mitochondrial energy production and comprises two functional domains: F1 and F0. The F1 domain contains five subunits (*α*, *β*, *γ*, *δ*, and *ε*), while the F0 domain consists of subunits a, b, c, d, e, f, g, I, k, A6L, F6, and the oligomycin-sensitive protein (OSCP) (108). Subunit, an and A6L of the F0 domain, are encoded by the *MT-ATP6* and *MT-ATP8* genes in mtDNA, while the remaining subunits, along with assembly factors (ATP assembly factors 1 and 2), coupling factors (inhibitor IF1 and coupling factor B), and ATP synthase-related proteins (MLQ and DAPIT), are encoded by nDNA (109). Mutations in *MT-ATP6*, *MT-ATP8*, *ATPAF2*, *TMEM70*, and *ATP5E* can lead to complex V deficiencies.

*MT-ATP6* mutations are the most common and widely studied causes of complex V deficiency, characterized by a reduction in ATP synthesis and basal oxygen consumption. This can lead to clinical manifestations such as ataxia, cognitive impairment, neuropathy, and epilepsy, with an incidence of 37%. MILS and NARP syndrome are associated with seizure rates of 86 and 44%, respectively, with generalized seizures being the most frequent type (110). Common mutations in *MT-ATP6*-related epilepsy include m.8993 T > G, m.8993 T > C, m.9032 T > C, m.9058A > G, and m.8921G > A (111–114). The m.8993 T > G mutation is particularly associated with a high incidence of generalized tonic–clonic, myoclonic, and progressive myoclonic seizures. EEGs in patients with this mutation typically show slow background activity and widespread sharp-slow complexes, making EEG a key tool for assessing disease severity In contrast, the m. 8,993 T > C mutation generally leads to milder symptoms and later onset, though it is still associated with generalized tonic–clonic seizures. Patients with the m.9032 T > C mutation may experience focal seizures, and EEG findings often show sharp waves in the central parietal region (115).

Mutations in MT-ATP8, such as m.8502A > T and m.8411A > G, have been identified in patients with epilepsy (116, 117). The former is closely associated with medial temporal lobe epilepsy, while the latter is linked to generalized tonic–clonic seizures. Mutations in *ATP5F1E*, *ATPAF2*, *ATP5PO*, and *TMEM70*, all encoded by nDNA, are also implicated in seizure disorders (108, 118, 119). Homozygous mutations in ATP5F1E (c.35A > G), which affect the *ε* subunit of the F1 domain, commonly result in generalized tonic–clonic seizures. A patient with an *ATP5PO* mutation encoding OSCP recently died from refractory status epilepticus, and a separate case involving a *TMEM70* mutation also presented with status epilepticus (119).

Treatment for complex V deficiency is largely symptomatic, focusing on the use of coenzyme Q10, vitamin B1, and vitamin C. *α*-ketoglutarate and aspartic acid have shown potential in increasing mitochondrial substrate phosphorylation, thereby normalizing ATP levels in cells with the m.8993 T > G mutation and reducing cell death (120). N-acetylcysteine and dihydrolipoic acid also hold therapeutic potential for patients with the m.8993 T > G mutation (121).

### 8.6 Coenzyme Q10 and epilepsy

Coenzyme Q (CoQ or ubiquinone) plays a crucial role in mitochondrial electron transfer, shuttling electrons from complex I or II to complex III. Coenzyme Q deficiency can be classified into primary and secondary forms based on etiology. Secondary CoQ deficiency is not directly related to the biosynthesis of CoQ, while primary CoQ deficiency results from mutations in genes responsible for CoQ biosynthesis. The Clinical manifestations are highly heterogeneous (122).

The CoQ biosynthesis pathway is complex and involves at least 15 genes, including *PDSS1*, *COQ1-PDSS2*, *COQ2*, *COQ3*, *COQ4*, *COQ5*, *COQ6*, *COQ7*, *COQ8-ADCK3*, *COQ8-ADCK4*, *COQ9*, *COQ10a*, *COQ10b*, *FDX1L*, and *FDXR*. Additionally, three other genes, *ADCK1*, *ADCK2*, and *ADCK5*, are also believed to play roles in CoQ biosynthesis (123). The incidence of primary CoQ10 deficiency is estimated at approximately 1 in 50,000, and while it can occur at any age, it is more common in children. When the central nervous system (CNS) is involved, the clinical phenotype is diverse, ranging from growth retardation and encephalopathy to intellectual disability and epilepsy. Several CoQ biosynthesis genes have been implicated in epilepsy including *COQ1-PDSS2*, *COQ2*, *COQ4*, *COQ5*, *COQ6*, *COQ8-ADCK3*, *COQ8-ADCK4*, and *COQ9*, with *COQ2*, *COQ4*, and *COQ8-ADCK3* mutations being more frequently associated with seizures (124).

*COQ4* mutations have the highest incidence of epilepsy, reaching up to 69%, with a higher prevalence among females. The age of onset ranges from birth to 9 years, and clinical symptoms may appear as early as the first week of life. These symptoms include growth retardation, epilepsy, skeletal muscle involvement, and cardiomyopathy (125). Seizure types include status epilepticus, myoclonic seizures, and focal seizures, with or without altered consciousness (126). EEG findings typically show reduced background activity and focal seizures, with occasional sharp waves, particularly in the left parietal region during REM sleep (127, 128).

Mutations in the COQ8A gene (also known as CABC1 or ADCK3) represent the most common form of primary coenzyme Q10 deficiency. Clinical manifestations include early-onset cerebellar ataxia, various motor disorders, cognitive impairment, motor intolerance, and epilepsy, which occurs in approximately 32% of cases. Epilepsy phenotypes associated with COQ8A mutations include focal seizures, generalized tonic–clonic seizures, myoclonic seizures, absence seizures, persistent partial epilepsy, and status epilepticus (129–132). EEG findings typically show frequent focal epileptic discharges and *δ* waves. During the interictal period, multi-spike wave or spike–wave rhythms are observed in the bioccipital region but may also be seen in the parietal, temporal, and frontal lobes. Some patients exhibit paroxysmal discharges in response to medium- and high-frequency (10-30 Hz) light stimulation (133). Cranial MRI frequently reveals cerebellar atrophy, though other regions may also be affected, including the brainstem and supratentorial areas such as the parietal and frontal insular lobes. Additionally, T2-weighted imaging often shows high signal intensity in the dentate nucleus and dorsal pons (129).

Primary CoQ deficiency was initially reported in association with COQ2 mutations, characterized by encephalopathy, cerebellar dysplasia, and epilepsy, with a seizure incidence of approximately 40%. Seizure types in COQ2 deficiency include focal seizures with altered consciousness, persistent partial seizures, myoclonic seizures, and status epilepticus (122, 134, 135).

Therapeutic response to coenzyme Q10 or its derivatives varies by genetic mutation. Patients with *COQ4* mutations may benefit from coenzyme Q10 supplementation, while those with *PDSS2*, *COQ8A*, and *COQ9* mutations tend to respond less effectively. For patients with COQ2 or COQ6 mutations, supplementation with 4-hydroxybenzoic acid (4-HBA) may enhance endogenous CoQ10 synthesis (136, 137). In preclinical studies, the antioxidant probucol has shown greater efficacy in treating renal and metabolic complications in *PDSS2* mutant mice but compared to coenzyme Q10 supplementation (138), this treatment remains under investigation.

### 8.7 Cytochrome C and epilepsy

Cytochrome c (CytC) is a water-soluble protein encoded by Ndna and composed of 104 amino acid residues. Its primary function is to facilitate electron transport between complex III and complex IV in the mitochondrial electron transport chain. Additionally, CytC plays a crucial role in apoptosis induction (139). During seizures, the production of harmful substances in neurons can increase the permeability of the mitochondrial inner membrane. CytC is subsequently released into the cytoplasm, where it binds to apoptosis-related factor 1(Apaf-1), promoting the formation of apoptotic bodies by binding with caspase-9. This process activates other caspases, leading to neuronal apoptosis. Mitochondrial damage exacerbates the susceptibility to seizures, creating a vicious circle that promotes further neuronal damage (140). Luan et al. found that a ketogenic diet can reduce the release of cytochrome c, thus playing a neuroprotective role (141).

## 9 Summary and prospects

When we encounter children with mitochondrial disease, it is necessary to conduct a series of relevant medical examinations. These auxiliary examinations that help diagnose mitochondrial diseases mainly include serum lactate levels, neuroelectrophysiological analysis, imaging diagnosis, and pathological examinations of muscles and brain, as well as diagnosis of gene mutations. Given that there is currently no definite treatment for mitochondrial disease, symptomatic treatment is particularly critical for patients. Some drugs may cause abnormalities in mitochondria or energy metabolism, so special care should be taken when using them. For example, sodium valproate may have significant side effects on the liver, so patients with mitochondrial disease should use it with caution. Although most of the drugs are still in development and trials, we outline in Table 2 the various modification therapies for mitochondrial diseases, such as small molecule therapy, gene therapy, and reproductive therapy.

**Table 2:** Disease-modifying treatments for mitochondrial diseases.

Methods		Drugs	Indications	Mechanisms	References
Symptomatic treatment		Control of epilepsy, control of blood sugar, treatment of acidosis, management of heart damage, management of gastrointestinal symptoms, control of lung infections, etc. may all be life-saving treatments for patients.			
Cocktail therapy		Combination of enzyme cofactors, antioxidants, amino acids and other nutritional supplements			
Small molecular drugs	Enhancing mitochondrial biogenesis	Bezafebrate	Mitochondrial myopathy	Activate PPAR which activates PCG-1α pathway	(160)
		5-Aminoimidazole-4-carboxamide ribonucleotide (AICAR)	Mitochondrial disease	Increase the activity of AMPK which activates PCG-1α by phosphorylation.	(161)
		Resveratrol	Mitochondrial myopathy disorder	An activator of AMPK and SIRT1	(162)
		RTA 408	Mitochondrial disease	Activate Nrf 2, which is PGC-1α downstream effectors	(163)
		Omaveloxolon (SKYCLARYS)	Friedreich’s ataxia	Nrf2 degradation inhibitor	(163)
	Restoration of the cellular NAD+ to NADH ratio	Nicotinamide Riboside (NR)	Mitochondrial myopathy disorder	Precursor of Nicotinamide adenine dinucleotide (NAD+)	(164)
		Nicotinamide mononucleotide (NMN)	_	Increase the cellular load of NAD through supplementation with precursors for de novo biosynthesis	(165)
		Acipimox (5-carboxyl-2-methyl pyrazine 1-oxide)	Mitochondrial myopathy	Lower plasma free fatty acids by inhibiting lipolysis through indirectly inhibiting hormone-sensitive lipase. This in turn promotes an increase in NAD+, sirtuin 1 (SIRT1) activation and enhanced mitochondrial gene expression	(166)
		Niacin	Mitochondrial myopathy; NAD+ deficiency	_	(167)
		ACMSD inhibitors	_	NAD booster concept	(168)
		Bacterial lactate oxidase fusion protein	_	Reoxidize extracellular lactate back to pyruvate, which is then transported back into the cell by the monocarboxylate carrier, allowing re-reduction of the pyruvate by lactate dehydrogenase to re-establish the NAD poise.	(169)
	Inducing Mitochondrial Turnover	Rapamycin	_	Target a component of the mammalian target of rapamycin (mTOR) complex, mTORC1, which is a key regulator of cellular homeostasis and has been linked to activation of the mitochondrial stress response in mitochondrial myopathy	(170)
	Increasing Mitophagy	urolithin A	_	A mitophagy activator	(171)
	Mitigating oxidative stress	Raxone (Idebenone)	LHON; Direct treatment of complex 1 defect	Idebenone is an analog of CoQ10 but has a higher, which can complex 1 deficiency in patients with LHON by directly transferring electrons to the complex 3, bypassing complex 1.	(172)
		Vatiquinone (EPI-743)	Friedreich ataxia; Mitochondrial disease with refractory epilepsy; Mitochondrial respiratory chain diseases	Inhibiting 15-lipoxygenase (15-LO) leads to increased GSH levels and decreased oxidized GSH	(161)
		RP103 (cysteamine bitartrate delayed-release capsules)	Mitochondrial disease	Cysteamine can increase intracellular glutathione levels by increasing the cysteine available for reduced glutathione synthesis	(165)
		Elamipretide (SBT-272)	Barth syndrome; primary mitochondrial myopathy	Cardiolipin protector; Inhibit cytochrome c peroxidase	(173)
	Restoring mtDNA Homeostasis	Nucleoside therapies [oral deoxypyrimidine (MT1621)]	Myopathic MDDS caused by TK2 deficiency	_	(174)
		Deoxynucleoside therapy	_	_	(175)
Gene therapy (Manipulating the Mitochondrial Genome)	Adeno-associated virus (AAV)		Barth syndrome, Friedreich ataxia, NDUFS4, NDUFS3, and SURF1-related Leigh syndrome, ethylmalonic encephalomyopathy, three mitochondrial DNA depletion disorders (mitochondrial neurogastrointestinal encephalomyopathy (MNGIE), MPV17 deficiency, and TK2 deficiency), Leber hereditary optic neuropathy and SLC25A46-related neuropathy.	Delivers therapeutic DNA to the cell nucleus, providing long-term gene expression with low immunogenicity, low risk of insertional mutagenesis, and the ability to deliver terminally differentiated cells	(170, 176)
	Antisense oligonucleotides (ASO)		ASO might be a viable therapeutic option for some PMDs, including a subset of POLG-related diseases	Selective degradation of target RNA and enhancement of translation	(177)
	Genome Editing	Mitochondrial-targeted restriction endonucleases (mitoREs)	These techniques have been tested exclusively in *in vitro* and *in vivo* preclinical models		(178)
		Zinc finger endonucleases (mitoZFNs)			
		Transcription activator-like effectors nucleases (mitoTALENs)			
		Meganucleases (mitoARCUS)			
		DdCBEs and TALEDs			
Reproductive therapy (prevent transmission of mtDNA mutations)	Mitochondrial Replacement techniques (MRT)	Pronuclear transfer (PNT)	For the treatment of unborn children whose parents have mitochondrial disease	The woman’s abnormal mitochondrial DNA is replaced with DNA from a healthy donor, or genetic material is transferred from a damaged oocyte into an enucleated donor cell.	(179)
		Maternal spindle transfer (MST)			
		Polar body transfer (PBT)			
		first polar body (PB1) and second polar body (PB2) transfer techniques			
	Targeted Genome Editing	RNA-free programmable nucleases		Mammalian mitochondria cannot repair double-strand breaks (DSBs) and degrade double-stranded linear DNA. Therefore, nucleases can be used to eliminate mtDNA.	(180)
		RNA-free DddA-derived cytosine base editors DddA-derived c ytosine base fusion editor		DddA-derived cytosine base editors (DdCBEs) allows C-to-T (or G-to-A) base editing	
	MRT and mtDNA editing combination therapy	_		MRT-Reconstituted oocytes or zygotes could be subjected to mtDNA editing to reduce or eliminate carried-over mutant maternal mtDNA.	(181)
Others	Exercise and endurance training	Mitochondrial disease	_	Enhance mitochondrial biogenesis	(182)
	Hypoxia	Mitochondrial disease	Vivo murine models of Leigh syndrome (Ndufs4 KO)	Hypoxia not only can trigger innate adaptive processes, but also limits the accumulation of toxic oxygen substrates.	(183)

The pathogenesis of childhood mitochondrial-associated epilepsy is highly complex, involving a range of genetic mutations that lead to various seizure types, often resulting in drug-refractory epilepsy. As research in mitochondrial-associated epilepsy advances, new gene mutations continue to be discovered. While significant progress has been made in understanding some aspects of the condition, many questions remain unanswered. The advent of next-generation sequencing (NGS) and the growing focus on precision medicine has facilitated the identification of mitochondrial genes linked to epilepsy. These discoveries have become integral to diagnosing hereditary epilepsy and have advanced the fields of personalized medicine and genetic counseling.

Currently, there are no effective treatments to completely cure mitochondrial diseases, and gene therapy remains in the experimental stages. However, both gene therapy and cell therapy are promising areas of research, offering potential for new and effective treatments. There is hope that future advancements in these therapies may ultimately lead to a cure for mitochondrial diseases.
